# Rapid Relief of Intractable Hiccups Through Bedside Diagnosis of Supragastric Belching: A Case Report

**DOI:** 10.1002/ccr3.70786

**Published:** 2025-08-05

**Authors:** Takeshi Ueda

**Affiliations:** ^1^ Department of Emergency and General Internal Medicine Rakuwakai Marutamachi Hospital Kyoto Kyoto Prefecture Japan

**Keywords:** breathing exercises, eructation, hiccup, physical examination

## Abstract

In patients with persistent hiccups, the presence of belching suggests an upper gastrointestinal cause. If symptoms resolve with open‐mouth breathing at the bedside, supragastric belching may be noninvasively suspected; allowing effective behavioral treatment and potentially reducing the need for further investigations.

## Graphical abstract

A stepwise bedside approach to diagnosing supragastric belching (SGB) in a patient with intractable hiccups.

(1) Persistent hiccups have a wide range of possible causes.

(2) When accompanied by belching, an upper gastrointestinal origin should be considered.

(3) If symptoms resolve with open‐mouth breathing, supragastric belching (SGB) is strongly suspected.

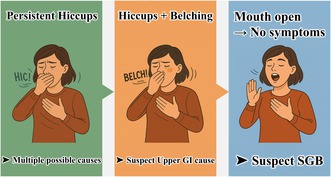

## 1 Introduction

Intractable hiccups, defined as those lasting more than 1 month [1], can be associated with various conditions, including central nervous system disorders, thoracic diseases, abdominal conditions, and metabolic disturbances [2].

Supragastric belching (SGB), a behavioral phenomenon, has been reported as a potential cause of intractable hiccups [3, 4]. SGB is characterized by the rapid ingestion and immediate expulsion of air from the esophagus, without the air ever reaching the stomach. This mechanism contrasts with that of gastric belching, in which air that has entered the stomach is released during a transient relaxation of the lower esophageal sphincter.

While SGB has been primarily studied in the context of gastroesophageal reflux symptoms, its role in triggering persistent or intractable hiccups remains under‐recognized.

SGB also differs from aerophagia, in which large volumes of air are swallowed and reach the stomach, leading to abdominal distention and discomfort. In contrast, the air in SGB is expelled before reaching the stomach; typically resulting in no abdominal distention.

Accurate differentiation between gastric belching and SGB generally requires esophageal impedance monitoring [5], a test that is not readily available at the bedside. However, if SGB‐induced intractable hiccups could be identified through bedside examination, the diagnostic process would be greatly simplified, avoiding the need to investigate intracranial, intrathoracic, or subdiaphragmatic causes.

Although no prior reports have described bedside diagnosis of SGB‐induced intractable hiccups, we present a case in which such a diagnosis was made based on physical examination findings, highlighting the potential utility of a simple bedside approach.

## 2 Case History and Examination

A previously healthy 27‐year‐old woman presented with persistent hiccups lasting 1 month. She initially sought care at another hospital, where an upper gastrointestinal endoscopy was performed because of accompanying heartburn. The findings were consistent with gastroesophageal reflux disease (GERD), and proton pump inhibitor (PPI) therapy was initiated. Although the heartburn resolved, the hiccups persisted for 6 months, prompting referral to our hospital.

The hiccups occurred continuously throughout the day and were frequently accompanied by belching. They were not triggered by eating, positional changes, or sleep. The patient denied abdominal distention or discomfort. She was a nonsmoker and reported only occasional intake of carbonated beverages or alcohol.

Two months before symptom onset, she had relocated and was prescribed escitalopram for anxiety. Approximately 6 months later, she experienced another significant life event—marriage. Although escitalopram was initiated before the hiccups began, a medication‐related cause could not be excluded. The drug was eventually discontinued without symptom improvement.

On initial presentation to our hospital, the patient's vital signs were within normal limits. Physical examination revealed no notable abnormalities in the head, neck, chest, or abdomen. Neurological examination was unremarkable.

## 3 Differential Diagnosis, Investigations and Treatment

Given the resolution of heartburn following PPI therapy and the continued presence of hiccups, we considered that gastroesophageal reflux disease (GERD) alone was unlikely to explain the patient's symptoms. Furthermore, the patient had no history of weight gain, dietary triggers, or supine reflux symptoms that typically worsen GERD. The absence of these features prompted consideration of a broader differential diagnosis. Potential causes included central nervous system lesions, thoracic disorders, diaphragmatic irritation, metabolic abnormalities, drug‐induced etiologies, and psychogenic factors. However, the lack of neurological signs, a normal physical examination, and the absence of systemic symptoms made structural and metabolic disorders less likely. Although escitalopram had been initiated prior to symptom onset, its discontinuation yielded no improvement, making a medication‐related cause unlikely.

While investigating potential causes, empirical pharmacologic treatment was initiated. Baclofen was started at 15 mg/day and produced partial improvement, but symptoms later recurred. The dose was increased to 30 mg/day without additional benefit. Subsequently, metoclopramide was prescribed, but it was ineffective.

During observation at our hospital, frequent belching and hiccups were noted. The hiccups often occurred during or immediately after a belch, with the belch appearing to precede the hiccup. These symptoms were provoked during conversation with the physician but diminished when the patient herself was speaking or distracted.

Physical examination during these episodes revealed subtle but consistent findings: intermittent contractions of the neck muscles preceding each belch and hiccup, irregular breathing patterns, and the absence of swallowing or Valsalva‐like maneuvers. Notably, when asked to open her mouth, both hiccups and belching ceased abruptly. See Video S1. This effect reversed upon mouth closure. In addition, voluntary diaphragmatic breathing—placing a hand on the abdomen and slowly expanding it during inhalation—also suppressed symptoms.

These observations strongly suggested supragastric belching (SGB) as the underlying cause of the hiccups. Rather than pursuing further pharmacologic or invasive investigations, a behavioral approach was recommended. The patient was instructed to regularly practice open‐mouth breathing and diaphragmatic breathing exercises.

## 4 Conclusion and Results (Outcome and Follow‐Up)

Following the bedside diagnosis of supragastric belching (SGB), the patient was advised to regularly practice open‐mouth breathing and diaphragmatic breathing. These nonpharmacologic techniques were selected based on their immediate effectiveness in suppressing symptoms during the physical examination.

A follow‐up phone interview conducted 3 months later revealed that the patient's symptoms had improved considerably. Although the hiccups had not completely resolved, the patient reported that the breathing exercises were more effective than any previous pharmacological treatments. She was able to manage the symptoms better in daily life and experienced reduced distress in social settings.

Importantly, the patient expressed satisfaction not only with the reduction of hiccups but also with the enhanced understanding of her condition. This awareness appeared to alleviate her anxiety and contributed to sustained engagement with the recommended behavioral strategies.

## 5 Discussion

In this case, supragastric belching (SGB) was suspected based on physical examination findings, without advanced diagnostics. Intractable hiccups—defined as those persisting for more than 1 month—can result from a wide range of conditions, including central nervous system, thoracic, abdominal, and metabolic disorders [2]. SGB has been reported as a rare but treatable cause of intractable hiccups [3, 4]. Given the broad differential diagnosis of intractable hiccups, identifying clinical clues at the bedside can guide management and prioritize diagnostic strategies.

A key diagnostic clue in this case was the concurrent presence of belching and hiccups. Belching is rarely caused by intracranial pathology [6], and when observed together with hiccups, suggests an origin in the upper gastrointestinal tract. Gastroesophageal reflux disease (GERD) is a common cause of both symptoms [7] and was initially suspected here based on endoscopic findings. However, the patient's symptom profile—lack of abdominal distention, absence of nocturnal symptoms, and response to behavioral interventions—was more consistent with SGB.

SGB is closely associated with GERD [8] and is strongly influenced by psychological factors. Cognitive behavioral therapy (CBT), including diaphragmatic and open‐mouth breathing, has demonstrated efficacy in reducing symptoms [9]. In this case, both hiccups and belching were alleviated by these techniques, supporting the diagnosis of SGB.

While hiccups could theoretically induce SGB via glottic closure and negative esophageal pressure, the temporal sequence observed here—belching preceding hiccups—suggests the reverse. Moreover, simultaneous resolution of both symptoms with open‐mouth breathing supports a shared underlying mechanism. Prior studies have proposed that abrupt esophageal distension can trigger hiccups [4]; which may explain their co‐occurrence in SGB.

Clinical features further supported the diagnosis. The patient's symptoms were absent during sleep, suppressed by distraction and conversation [10, 11], and not associated with meals—typical characteristics of SGB.

SGB is thought to result from pharyngeal air propulsion and inspiratory negative pressure, both of which may produce characteristic physical signs [12]. Careful physical examination can reveal subtle findings suggestive of these mechanisms, including nonswallow‐related laryngeal movement, facial tension with tight lip closure before belching, and transient apnea following deep inspiration [10]. These signs, though easily overlooked, can assist in bedside diagnosis. Clinicians must distinguish them from swallowing or voluntary breath‐holding, such as in the Valsalva maneuver [13].

Although esophageal impedance monitoring is the gold standard for diagnosing SGB [14], it is rarely used in routine practice due to its complexity and cost. Therefore, simpler, effective bedside methods are needed. In this case, both open‐mouth and diaphragmatic breathing suppressed symptoms by preventing air entry into the esophagus. The rapid response to behavioral strategies, despite the failure of pharmacological treatments such as metoclopramide and baclofen [13], supports a true behavioral mechanism rather than a placebo effect alone; although the latter cannot be entirely excluded.

Beyond symptom control, the success of these breathing techniques had a positive impact on the patient's understanding of her condition, strengthening the therapeutic alliance and facilitating the introduction of CBT. The clinical response to CBT further reinforced the diagnosis of SGB and contributed to patient satisfaction.

## 6 Conclusion

This case highlights that supragastric belching (SGB) can be accurately suspected through careful bedside observation, even in patients presenting with intractable hiccups. Key clinical features—including belching preceding hiccups, absence during sleep, and suppression with open‐mouth and diaphragmatic breathing—enabled a behavioral management approach without resorting to invasive diagnostics in this particular case. These simple techniques contributed to both symptom relief and the early initiation of cognitive behavioral therapy. Recognizing SGB at the bedside may offer a practical and cost‐effective approach in the evaluation and management of intractable hiccups.

## Author Contributions

**Takeshi Ueda:** conceptualization, data curation, formal analysis, writing – original draft, writing – review and editing.

## Consent

Written informed consent for publication was obtained from the patient.

## Conflicts of Interest

The author declares no conflicts of interest.

## Supporting information


**Video S1:** Supragastric belching and hiccups improved by open‐mouth breathing. This video demonstrates irregular breathing and slight contractions of the neck muscles, followed by hiccups and subsequent supragastric belching. Both symptoms cease immediately upon opening the mouth; however, they recur when the mouth is closed. Notably, the symptoms are suppressed when the patient is focused on conversation with the physician.

## Data Availability

The author has nothing to report.
